# Host Generated siRNAs Attenuate Expression of Serine Protease Gene in *Myzus persicae*

**DOI:** 10.1371/journal.pone.0046343

**Published:** 2012-10-10

**Authors:** Varnika Bhatia, Ramcharan Bhattacharya, Prem L. Uniyal, Rajendra Singh, Rampal S. Niranjan

**Affiliations:** 1 National Research Centre on Plant Biotechnology, Indian Agricultural Research Institute Campus, New Delhi, India; 2 Department of Botany, University of Delhi, Delhi, India; 3 Phytotron Facility, Indian Agricultural Research Institute, New Delhi, India; Nanjing Agricultural University, China

## Abstract

**Background:**

Sap sucking hemipteran aphids damage diverse crop species. Although delivery of ds-RNA or siRNA through microinjection/feeding has been demonstrated, the efficacy of host-mediated delivery of aphid-specific dsRNA in developing aphid resistance has been far from being elucidated.

**Methodology/Principal Findings:**

Transgenic Arabidopsis expressing ds-RNA of *Myzus persicae* serine protease (MySP) was developed that triggered the generation of corresponding siRNAs amenable for delivery to the feeding aphids. *M*. *persicae* when fed on the transgenic plants for different time intervals under controlled growth conditions resulted in a significant attenuation of the expression of *MySP* and a commensurate decline in gut protease activity. Although the survivability of these aphids was not affected, there was a noticeable decline in their fecundity resulting in a significant reduction in parthenogenetic population.

**Conclusions/Significance:**

The study highlighted the feasibility of developing host based RNAi-mediated resistance against hemipteran pest aphids.

## Introduction

Loss in yield, especially due to insect pests, accounts almost 14% of global agricultural output [1] and incurs a cost of about 3000 million dollars towards the protection of five important crop species [2]. Among insect pests, sap sucking hemipteran aphid elicits multitude damaging effects on a large number of agriculturally important oilseed, cereal, fruit, timber and medicinally important crop species belonging to taxonomically diverse families including Poaceae, Anacardiaceae, Rosaceae, Brassicaceae, Pinaceae, and Asteraceae [3], [4]. For instance, sieve diversion by numerous aphid nymphs and adults render the plant completely devitalized resulting in barren inflorescence. Often photosynthesis of the infected plants is impaired due to the growth of saprophytic sooty mould on aphid honey dew. Furthermore, while feeding on the plant it also spreads luteoviruses accounting for about 45% insect-borne viruses [5], [6]. Since aphids feed through sucking the phloem sap and shelter beneath the leaf surface, only systemic chemical insecticides are effective against aphids. However, use of chemical insecticides results in inevitably high residual toxicity. The problem is further accentuated due to a rather limited variability among the crossable germplasms towards aphid resistance. As a result, breeding efforts for developing resistant cultivars for minimizing the use of insecticides has not met with any success. Therefore, it is imperative to exploit the potential of environmentally benign transgenic technology specific to the target group of insect pests. Although some efforts have been made towards developing transgenic plants expressing insecticidal proteins of plant origin such as lectins [7], [8] and protease inhibitors [9], [10], the success has been rather modest due to their non-specific mode of action.

RNA interference (RNAi) pathway, found ubiquitously across plant, insect and mammalian genera, plays a vital role in defending hosts against a wide spectrum of parasitic genes [11]–[14]. In insects, RNAi is usually accomplished by injection of custom-synthesized siRNAs [15], [16] or dsRNAs [17]–[19]. The cells of the insect gut lumen can also take up dsRNA for exerting the RNAi effects either by oral delivery through artificial diet [20], [21] or ingestion of bacteria expressing dsRNA [22], [23]. These and several other studies thus provide ample evidences toward the possible intervention of RNAi technology for pest control by knocking down vital insect genes [24], [25]. Among the insect pests, aphids also respond to the successful delivery of siRNA or dsRNA by triggering RNAi responses [15], [26]. However, the delivery of siRNA in aphids has been limited to microinjection and thus a bottleneck for its application in pest control. Although mechanistic details governing the uptake of dsRNA in aphids still remain elusive, the host-mediated delivery of dsRNA is still an attractive paradigm for developing aphid resistance. In insects, RNAi could be either cell autonomous or non-cell autonomous with the former limited to the cellular location of dsRNA application, whereas the latter spreads systemically across the cells different from the site of dsRNA application [23]. Systemic RNAi is considered to be mediated through a widely conserved transmembrane protein SID-1 to transport RNAi silencing signals between cells [27]. SID-1 appears to be conserved in many insect taxa except a few [28], [29]. Presence of SID-1 homologs in many of the aphid species therefore, suggests the likely occurrence of systemic RNAi [30]. However, incongruities were evident in this dogma from the studies that showed lack of systemic RNAi despite the presence of three orthologs of SID-1 in *Bombyx mori* and its prevalence in mosquito lacking these orthologs [17], [29]. Considering this impasse regarding the regulatory mechanisms governing the systemic RNAi, it is rather logical to target an aphid gene for which the site of function will be the same as the site of dsRNA or siRNA application.

Digestive proteolytic activity of aphid nymphs predominantly relies on abundantly present serine proteases [31]–[34]. Accumulation of protease inhibitors in the tissues under predation by insect is the major response of innate defence against herbivory in plants [35]. The transgenics expressing protease inhibitors have also shown deterrent effect against aphids in feeding trials [10], [36], [37,]. Gut lumen of aphids lined with perimicrovillar membrane (PMM) offers large absorption area for exogenously fed siRNA molecules. Therefore, transgenic host expressing dsRNA of serine protease gene could be an attractive and a viable proposition for developing aphid resistance.

Here, in the present study we demonstrate transgenic plant-mediated delivery of siRNA to elicit RNAi of the aphid-specific serine protease. The results provide empirical evidence towards attenuation of both the gene expression and enzyme activity of target serine protease in the aphids that fed on transgenic *Arabidopsis* expressing SP-bound siRNAs. The study highlights the potential efficacy of RNAi as a vital component of integrated pest management.

## Results

### Expression of Serine Protease in Green Peach Aphid and siRNA Target in the Transcript

**Figure 1:**
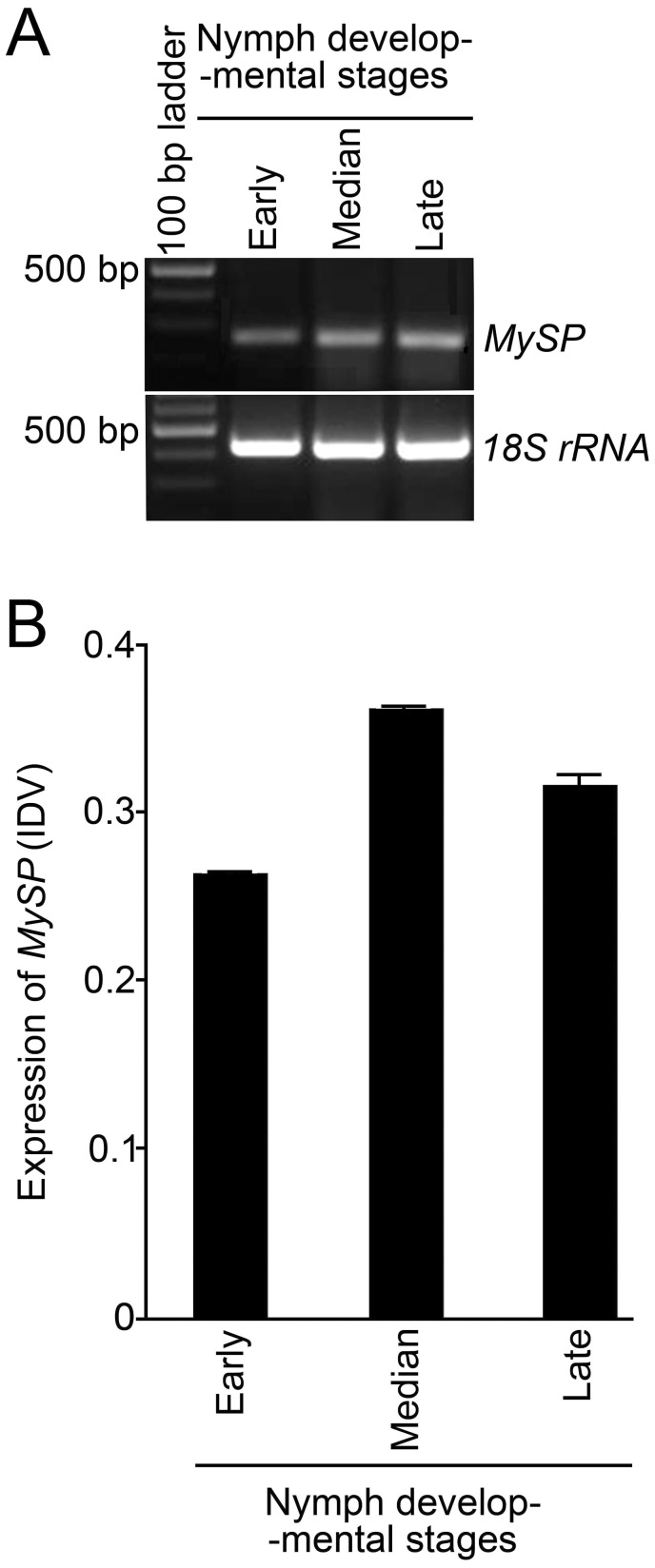
Expression analysis of *MySP* during nymph developmental stages of *M. persicae*. (A) Expression profiles of *MySP* at different developmental stages of *M. persicae* ranging from early (1–2 day-old new-born nymph), median (3–5 day-old nymph) to late (6–7 day-old apterous adult) was determined by semi-quantitative RT-PCR. cDNA, prepared from 5 µg of total RNA in each sample, was PCR amplified with *MySP* specific primers (lanes 2–4; upper panel) and 18SrRNA (lanes 2–4; lower panel). The latter was used as endogenous control showing similar RNA concentrations in aphid samples collected at different developmental stages. (B) Integrated density values (IDV) of the PCR products of *MySP* in the nymphs collected at different development stages as described in (A).

In aphids, newborn nymphs suck excess of phloem sap from the host plant and develop into a reproducing adult within about a week. Serine proteases, present across the insect species, play a pivotal role in gut proteolytic digestion [38]. Therefore, in the present study we hypothesized a likely influence of gene-specific siRNA on the relative expression profile of serine protease gene during the most damaging nymphal stage of green peach aphid (*Myzus persicae*). From here onwards *MySP* refers to the serine protease gene in *M. persicae*. Among aphid species, the sequence of serine protease gene is available in the database only for pea aphid (*Acyrthosiphon pisum*). Therefore, this sequence was used for designing a pair of heterologous primers for semi-quantitative RT-PCR analyses for determining the changes, if any, in the transcript levels of *MySP* during three developmental stages of the wingless nymphs representing early (1–2 days), median (3–5 days), and late (6–7 days) growth phases (Fig. 1). Differential expression of *MySP* (an amplicon of 239 bp) was revealed across these three developmental stages and amplification of 18SrRNA (an amplicon of 427 bp) was used as an internal control (Fig. 1A). The expression of *MySP* at three developmental stages was further quantified using integrated density values (IDV) of the PCR products (Fig. 1B). Although IDV values of *MySP* showed variations across different nymph development stages, no specific trend was evident. Nevertheless, the results clearly suggested that *MySP* transcripts in the wingless nymphs are amenable to siRNA-mediated knockdown at any of its three development stages categorized in the present study. Therefore, it was logical to assume that targeted silencing of this gene in the gut of the aphid would potentially have an adverse effect on its feeding ability and consequently on its fecundity and reproductive fitness.

### Sequence Analysis and Phylogenetic Lineage of *MySP*

**Figure 2:**
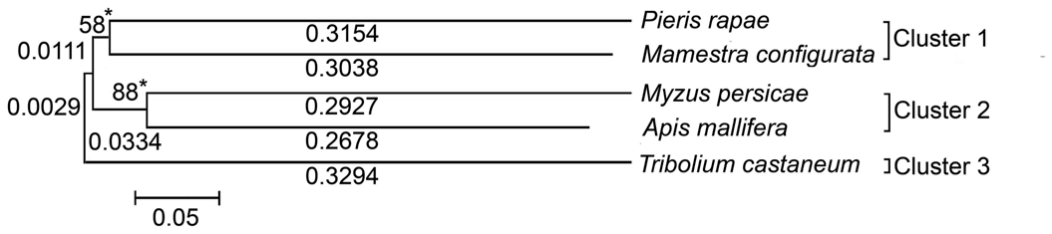
Dendrogram illustrating the relatedness of serine protease gene sequence of *M. persicae* (*MySP*) with those of different Indian pollinators. Phylogenetic relationship of *MySP* and its counterpart from Indian pollinators was conducted using MEGA 4.0.2 which is shown along with branch lengths. DNA sequences were aligned using CLUSTAL W and a tree was constructed by neighbour-joining program from a similarity matrix of pairwise comparisons. The Nucleotide/ p-distance option was selected in substitution model section. Bootstrap values (*) were assessed with 1000 replicates and are shown at the dendrogram nodes. The scale bar represents sequence divergence.

The *A. pisum* serine protease sequence was analyzed *in silico* for identifying potential sites for siRNA-mediated transcript knockdown. Nucleotide region between +276 to +826 bp was identified as potential site for the production of corresponding siRNA molecules. A homologous *MySP* gene sequence was PCR amplified from cDNA of *M. persicae.* The amplified *MySP* gene sequence showed 91% homology with its counterpart in *A. pisum.* To examine any homology between the targeted *MySP* sequence to its orthologous counterparts in beneficial pollinators, serine protease gene sequences of pollinator insects *Apis mellifera*, (Accession no. NM_001011584), *Tribolium castaneum* (Accession no. NM_001170771), *Mamestra configurata* (Accession no. FJ205442), and *Pieris rapae* (Accession no. FJ882067) from GenBank were aligned using Clustal W program for generating a phylogenetic tree (Fig. 2). The phylogenetic tree showed five serine protease sequences originating from three clusters of which two were branched. Cluster 1 included *P. rapae* and *M. configurata* with 61.92% divergence between them. In Cluster 2, *M. persicae* and *A. mellifera* evolved from a common ancestor but diverged 56.05% between them. Clusters 1and 2 diverged from the third cluster, comprising a single branch representing *T. castaneum,* by 34.34% and 36.57%, respectively. The evolutionary history was inferred using the neighbor-joining method (1000 bootstraps). The optimal tree with the sum total of 1.55 branch length is shown and different branch lengths are specified. The tree is drawn to scale, with branch lengths in the same units as those of the evolutionary distances used for inferring the phylogenetic tree. The bootstrap values are depicted at the node of the dendrogram. Codon positions included were 1^st^, 2^nd^, 3^rd^ and non-coding sites. All positions containing gaps and missing data were eliminated from the dataset. There were a total of 562 positions in the final dataset. In addition, all the four serine protease sequences of Indian pollinators when aligned individually with *MySP* sequence did not show any significant similarity. The phylogenetic analysis of *MySP* thus conformed to its specificity thereby predicting a minimal probability of affecting the serine protease of non-target insects.

### Suppression of *MySP* Expression and Protease Activity in SP-transgenic Fed *M. persicae*

**Figure 3:**
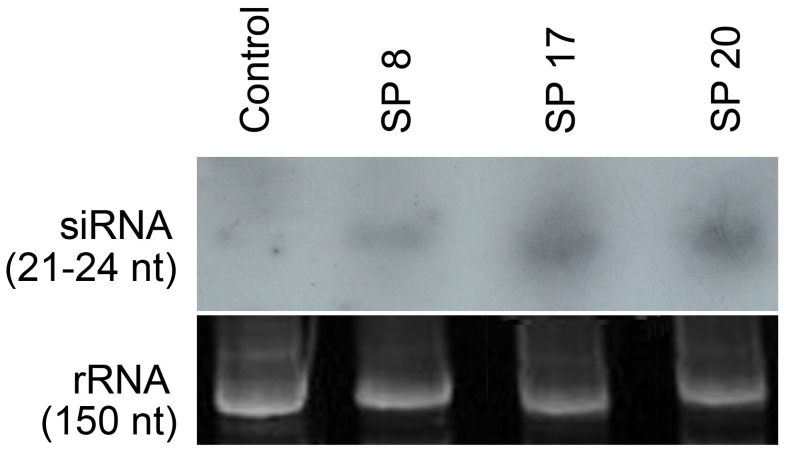
Detection of *MySP*-specific siRNAs in different SP transgenic lines. Total RNA (50 µg) was extracted from the control and different SP transgenic lines (SP8, SP17, and SP20), run on 15% denaturing acrylamide gel, and hybridized with *MySP*-specific RNA probes. Northern hybridization detected 21–24 nt siRNAs in transgenic samples (upper panel). Loading of equal amounts of RNA was confirmed by ethidium bromide staining (lower panel).

**Figure 4:**
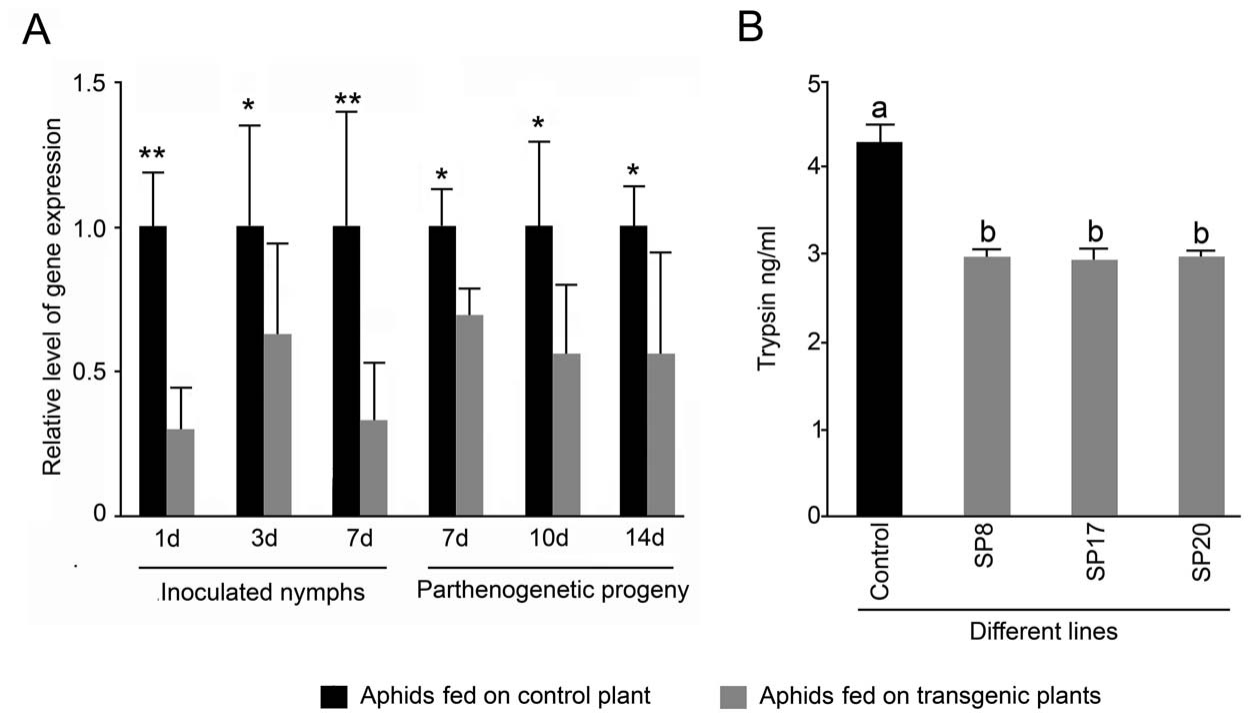
siRNA-mediated suppression of *MySP* transcript and gut protease activity in aphid nymphs fed on SP transgenic lines. (A) Aphids were collected from the control and SP-transgenic line at different time points after their release and also those developed parthenogenetically for quantitative Real-time PCR analysis of the relative expression profiles of *MySP*. 18S rRNA was used as an internal control, and values for the control plants (inoculated nymphs and parthenogenetic progenies) were normalized to 1. Data presented are means of two independent biological replicates with three technical replicates of each ± SD. Asterisks * and ** indicate reductions in the expression of *MySP* by ≤ 0.5 fold and > 0.5 fold, respectively in the aphids that were fed on the SP-transgenics as compared to those on the control plants. (B) Gut protease activity was estimated by EnzChek protease assay performed with 10 µg of total protein extracted from *M. persicae* collected from the control and SP transgenics 7 days after the release of the aphids. Protease activity was expressed as equivalent to trypsin (ng/ml). Values are mean ± SE; *n = *4. Different letters on the histograms indicate that the means differ significantly (*P* ≤ 0.05).

Several T_2_ homozygous lines of transgenic Arabidopsis lines expressing ds*MySP* (henceforth named as SP-transgenics) were recovered through segregation analyses of kanamycin resistance trait combined with *MySP* specific PCR and RT-PCR analysis. Plants transformed with empty vector were used as control for the ds*MySP* treatment. Semiquantitative RT-PCR using a forward primer targeted to the loop region of ds*MySP* along with the *MySP* reverse primer detected expression of ds*MySP* RNA in SP-transgenics (Fig. S2B). The control plants demonstrated expression of *nptII* but did not show any amplification for ds*MySP* primers. T3 plants from selected homozygous lines were grown to maturity to examine the effect of ds*MySP* on the feeding *M. persicae*. *In vitro* transcribed radio-labeled RNA probes corresponding to *MySP* sequence detected 21–24 nt siRNAs during northern blot analysis of the SP-transgenics (SP8, SP17, and SP20) (Fig. 3). The probe did not yield any hybridization signal in RNA samples of vector transformed control plants. The results clearly suggested the processing of the double stranded hair-pin loop structure of the *MySP* transcript into siRNAs in SP-transgenics. Further *M. persicae* nymphs (3–5 day-old), reared and maintained on wild type plants, were released to feed on SP-transgenic lines for monitoring the potency of SP-transgenics in attenuating the expression of *MySP* and protease activity in *M. persicae* nymphs during feeding at different developmental stages (Fig. 4). Real-time PCR analysis was carried out for measuring the relative levels of *MySP* transcripts in the nymphs that were collected at different time points (1, 3, and 7 day-old) after their release, and also from 7-, 10-, and 14-day-old parthenogenetically developed progenies *in situ* from the control and the SP-transgenic line (Fig. 4A). In order to ensure that there is no *MySP* amplification from any remnant of ingested ds*MySP* RNA during real time PCR, a forward primer, which binds outside the cloned *MySP* region, was used along with a reverse primer within the cloned sequence. The amplification plot and dissociation curve for *MySP* transcript demonstrated the proper amplification and no non-specific binding of primers. The *MySP* transcripts showed variable but significant reductions in the nymphs (inoculated and parthenogenetic progenies) across all the developmental stages when fed on SP-transgenic lines. However, no specific trend in the reduced expression of *MySP* in the nymphs could be established either with respect to the sample types (inoculated and parthenogenetic progenies) or with an increasing duration of siRNA ingestion. The primer pair used in qPCR analyses did not show any amplification with either cDNA from the transgenic leaves or plasmid DNA of the RNAi construct conforming to its specificity to aphid- MySP transcripts. Non-specific trend and also variations in the expression values of *MySP* in some of the nymph samples could be attributed to the fact that each nymph sample analysed from the control and SP-transgenic plant comprised many individual aphid nymphs which would have presumably varied in the amount of ingested sap from the control or SP-transgenic plant. Nonetheless, the results provided explicit evidence towards a rather unambiguous attenuating effect of siRNAs, expressing in SP-transgenics, on the expression of *MySP* in the nymphs representing various developmental stages. The result was also consistent with the detection of *MySP* expression during early (1–2 days), median (3–5 days) and late (6–7 days) growth phases of nymph (Fig. 1).

To further determine whether attenuation in the expression of *MySP* in the nymphs that were fed on SP-transgenics elicited any commensurate effect on its digestive ability, the gut protease activity was quantified (Fig. 4B). Gut protease activity of aphids fed on SP-transgenics was measured by inherent ability of its protein extract to hydrolyze fluorescent labeled casein that was quantified as trypsin equivalent by extrapolating the experimental values on a standard trypsin digestion curve. As was anticipated, there was a significant (*P<0.05*) decline (∼ 31%) in the protease activity of the 10–12 day-old nymphs that were fed on SP-transgenics for 7 days compared to those allowed to feed on control plants.

### Expression Analyses of Off-target Genes in *M. persicae* Fed on SP-transgenics

Non-specific attenuating effect of MySP-siRNAs, if any, on the expression of genes that may have some degree of sequence similarity with *MySP* was examined. To achieve this, pairwise local alignment of *MySP* and several putative *Acyrthosiphon pisum* gene sequences using ‘EMBOSS Water tool (http://www.ebi.ac.uk/Tools/psa/) was employed. The analysis revealed two *A. pisum* mRNA sequences i.e., *ApSP1* (Acc. No. NM_001162294.1) and *ApSP2* (Acc. No. NM_001161907.1) that showed maximum possible similarity of 41.1% and 40.3% respectively, to *MySP*. In off-target analysis for amplification of their respective counterparts in *M. persicae* RNA, heterologous sequence information of *ApSP1* and *ApSP2* was used for designing the primers. Real-time PCR analysis was carried out for measuring the relative transcript levels of two MySP-like sequences *ApSP1*, *ApSP2* and other *M. persicae* genes belonging to different functional categories i.e., *OBP* (odorant-binding protein, Acc. No. FJ387486.1), *CSP1* (chemosensory protein, Acc. No. FJ387490.1) and *ACE* (acetylcholine esterase, Acc. No. AF287291.1) in the nymphs that were collected at different time points of feeding on the control and the SP-transgenics (Fig. S3). The amplification plot and the dissociation curve for each of the target transcripts demonstrated proper amplification and specificity in primer binding. However, no significant differences were detected in the relative transcript levels of any of these off-target genes in the aphids that were fed on either control or transgenic plants. The results thus suggested potential target specificity of *MySP*-siRNAs.

### Attenuation of Fecundity in Aphids Fed on SP-transgenics

**Figure 5:**
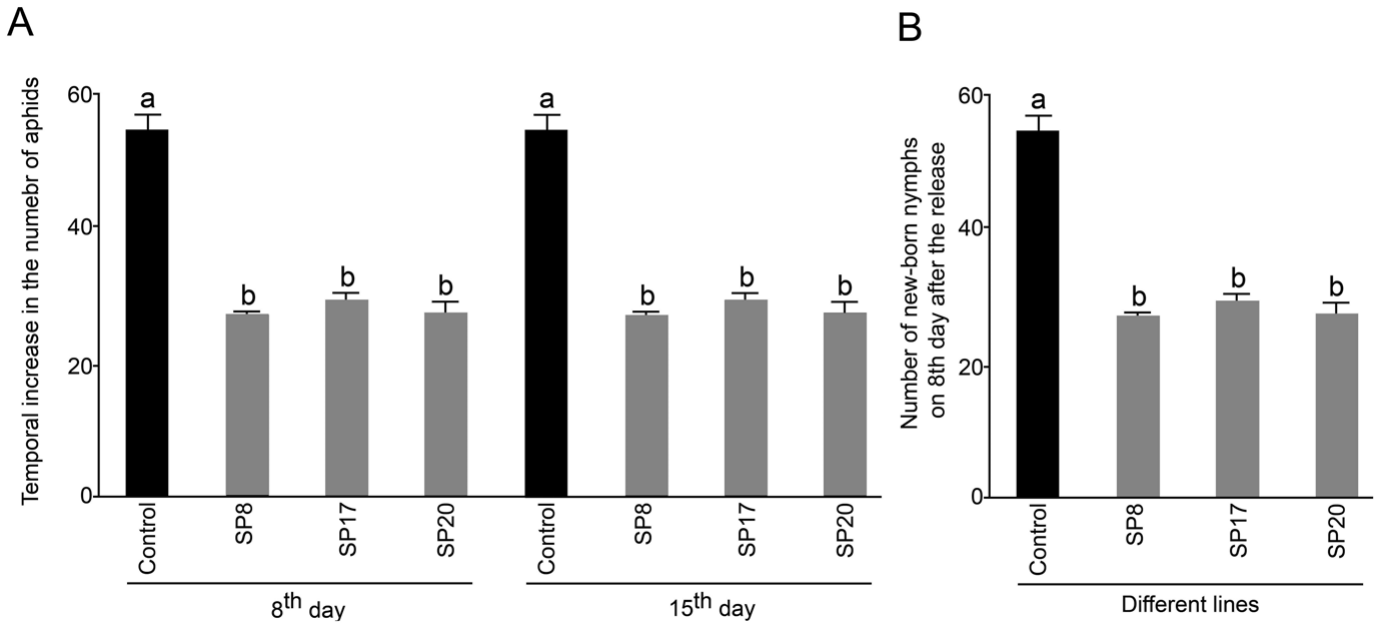
Population increase and fecundity of aphids released on control and *MySP*-silenced transgenic lines. Nymphs (3–5 day-old) of *M. persicae* were released on 21day- old control and SP-transgenic plants (SP8, SP17, and SP20). The inoculated plants were reared in a growth chamber under controlled conditions (24°C ± 2°C, 80–85% relative humidity and a 16 h light / 8 h dark photoperiod). Data are presented for the control and SP transgenics showing (A) mean increase in the total number of aphids (counted manually) on 8^th^ and 15^th^ day after their release, and (B) the fecundity rate of aphid nymphs. Values for (A) are mean ± SE; *n = *6, whereas for (B) it represent average number of nymphs produced per 10 aphids ± SE; *n = *3. Different letters on the histograms (A and B) indicate that the means differ significantly (*P* ≤ 0.05).

The effect of attenuated expression of *MySP* transcripts, if any, on the survivability of SP-transgenics fed aphids was assayed (Fig. 5). In about 8 days, released nymphs matured into adults and started producing new nymphs on both the control plants and SP-transgenics. No significant (*P* <0.05) differences were evident on the mortality rate even after 15 days of their release on control and SP-transgenic plants (data not shown). Interestingly though, there was a substantial reduction (about 50%) in the total number of aphids on SP-transgenic plants (SP8, SP17, and SP20) recorded on 8^th^ and 15^th^ day after their release compared to those on the control plants (Fig. 5A). Likewise, there was about 50% reduction in the number of new-born nymphs on SP-transgenic plants on 8^th^ day after insect release compared to those on the control plants (Fig. 5B). The results explicitly demonstrated the suppression of parthenogenetic fecundity in aphids when fed on SP-transgenic plants. A similar decrease in fecundity was consistently observed on 15^th^ day after the insect release.

## Discussion

Bioinformatic analysis revealed 18 candidate gene sequences putatively coding for serine proteases in *Acyrthosiphon pisum* (Fig. S1). However, for *Myzus persicae*, the availability of gene sequences is limited in public domain database and no sequence was available that codes for serine protease. In the present study, to isolate *MySP,* XM_001951259.1 was selected largely because it did not show any significant homology with other members of this gene family, and along with *MySP* it represented a distinct clad in the phylogenetic tree. Serine proteases are ubiquitous in animals and in other insect species they are present as gene families with large number of homologs [39], [40]. *M. persicae* feeds on hundreds of host plants belonging to more than 40 different plant families including Solanaceae, Chenopodiaceae, Asteraceae, Brassicaceae, Cucurbitaceae etc. Beneficial pollinators viz. *Pieris rapae* and *Mamestra configurata* occur on members of the family Brassicaceae while *Apis mellifera* on Asteraceae members along-with *M. persicae*. It is presumed that siRNAs may be transferred to the pollinators when they suck the plant sap. Interestingly, phylogenetic analyses of selected *MySP* sequence revealed stretches of nucleotide sequences that were non-overlapping to the corresponding gene of any of the beneficial pollinators. This suggested a likelihood of low risk of siRNAs, targeted to the non-overlapping stretch, of affecting non-target insects. Serine proteases are known for their function in digestion, embryonic development and defense responses of insects challenged by microbial and parasitoid wasp invaders [41], [42]. Semi-quantitative RT-PCR revealed significant accumulation of *MySP* transcripts irrespective of various developmental stages of wingless nymphs (Fig. 1). This highlighted the potential role of *MySP* during different stages of growth and development. However, in aphids none of the members of protease gene family including *MySP* has been functionally characterized. Transgenic expression of protease inhibitors for suppressing insect proteases has been a method of choice for reducing the performance of sap sucking insect pests including aphids in transgenic plants [10], [43]. Unlike transgenic expression of inhibitor to protease, the biggest advantage of RNAi- mediated suppression is its high level of specificity towards target insects. Even closely related species can be selectively killed by feeding on dsRNAs that target the more variable regions of genes [44].

Earlier studies have demonstrated successful expression of dsRNA in leaf phloem by the constitutive CaMV35S promoter [45]. Presence of inverted repeat-transcript in Arabidopsis can trigger the plant dicer DCL4 to process it into siRNAs [46]. Northern hybridization of the SP-transgenics demonstrated plant dicer-mediated processing of *MySP* transcript into 21–24 nt siRNAs which are known to be mobile in plants [47]. The siRNAs on being ingested through plant sap attenuated endogenous serine protease transcript of the feeding aphid population. However, attempts to detect MySP-siRNAs in aphids fed on SP-transgenics through northern hybridization of total RNA as well as enriched small-RNAs did not succeed. Enrichment of target siRNAs using target tissue as the source material for small-RNA isolation could possibly overcome this technical limitation. The qRT-PCR data showed that the inhibition started after day 1 of aphid release and continued at variable level throughout the different developmental stages of nymphs. In pea aphid, transcript down regulation began one day after the microinjection of cathepsin-L specific dsRNA and stopped at 7 days [26]. Our result indicates that transgenic mediated delivery system is able to initiate transcript attenuation with same rapidity as direct microinjection. In persistent effect of RNAi, the temporal small RNAs are amplified by a host RdRp. In insects, presence of RdRp ortholog was never confirmed [48]. In the absence of RdRp mediated amplification of ds-RNA, host mediated delivery ensures a continuous delivery of siRNA and persistent RNAi effect in contrary to single shot microinjection in which the inhibitory effect stopped at 7 days onwards [26]. We quantified 27–74% transcript down regulation similar to that observed in case of microinjection as well as artificial diet mediated delivery against aphids [44], [49]. A recent attempt on host mediated delivery of siRNA against *M. persicae* also, demonstrated down regulation of the target transcripts up to 60% [50]. *MySP* sequence did not show significant homology either to any of the SP-homologs or other off-target aphid genes. Therefore, no attenuating effect was anticipated on the expression of any of the off-target aphid genes. qRT-PCR based transcript analyses for *MySP*-like sequences (*ApSP1* and *ApSP2*) and other vital aphid genes (*OBP*, *CSP*, and *ACE*) in control-fed and transgenic-fed aphids confirmed target specificity of *MySP*-siRNA and complete absence of any off-target effect.

Microinjection of 276 ng ds-COO2 /aphid conferred significant insect lethality in *A. pisum* [26]. Likewise, ds-RNA-mediated perturbation in vATPase gene expression triggered lethal responses in different species of *Drosophila* [44]. We recorded significant decrease in fecundity of SP-transgenic fed aphids (Fig. 5B). Fecundity was reduced by almost 50% in transgenic fed aphids compared to the control. However, we did not observe any insect mortality. This is in contrast to the results obtained in feeding [44] and microinjection [26] of dsRNA in *A. pisum*. This is probably due to the reason that attenuation of *MySP* transcripts reduced gut protease activity, albeit not completely and there are alternate proteases present in aphid gut viz. cysteine proteinases, aspartic proteinases, metalloproteinases [51]. Our result is also in agreement with the observation reported by Pitino, et al. [50], when *M. persicae* nymphs were fed on transgenics generating *Rack-1* and *MpC002* siRNAs. Reduced fecundity has also been reported as effect of lectin transgenics on aphids [8], [52] and discussed as potential mechanism to control aphid population build up. In our observations even within first two weeks of infestation the population build up in transgenics was two-fold less compared to non RNAi plants. Therefore, the results provide empirical evidence to the possible application of RNAi as a control strategy to aphid infestation. In perspective to further application of this technology, it is pivotal to select appropriate aphid gene as target for knockdown. Present limitation of paucity in known aphid genes is soon going to be overcome as sequencing of *M. persicae* genome is under progress (HGSC, Baylor College of Medicine, Texas, USA) and *A. pisum* genome sequence is already available [53], [54].

Aphid with intrinsic capacity of rapid evolution, insect population developing resistance against a particular ds-RNA sequence cannot be ignored. However, management strategy for sustainability of dsRNA pesticides seems to be less difficult. The situation can be dealt either by deploying different dsRNA region within the same target gene or taking a different target gene. Resistance development due to mutations in RNAi machinery is less likely as the RNAi pathway is also involved in endogenous gene regulation of the organisms [55].

## Materials and Methods

### Plant and Insect Maintenance

The wild type and the transgenic *Arabidopsis thaliana* cv. Columbia plants were grown to flowering stage and maturity in transgenic glasshouse facility of NRC on Plant Biotechnology, New Delhi under controlled growth conditions (24 ± 2°C, 80–85% relative humidity and diurnal rotation of 16 h light / 8 h dark). Single clone of aphid species was identified as *M. persicae* by the Entomology Department, Punjab Agricultural University, Ludhiana, Punjab. For continuous culture of *M. persicae*, nymphs were reared on 3–4weeks grown Arabidopsis plants. At every three weeks interval fresh plants were infected with *M. persicae* nymphs and older infected plants were discarded.

### Expression Analysis of *MySP* Gene in *M. persicae*

*Myzus persicae* reared on Arabidopsis were harvested and sorted into three growth phases viz. early (1–2-days), median (3–5-days), and late (6–7-days old apterous adults) growth phases. Aphid RNA was isolated using RNeasy Mini Kit (Qiagen, USA) and 5.0 µg of total RNA was converted into cDNA using Superscript III First-Strand Synthesis System (Invitrogen, USA). cDNA (2.5 µl) was PCR amplified with *MySP* gene-specific primers (SP_1F and SP_1R, Table S1) and 18S rRNA gene was amplified simultaneously as internal control to ensure equal amount of cDNA in each tube. The cycling profile comprised an initial denaturation at 95°C for 5 mins, followed by 30 cycles of 95°C for 30 sec, 55°C for 30 sec, 72°C for 1 min, and a final extension at 72°C for 5 min. The sequence was submitted to GenBank (GQ426953.1).

### *In silico* Identification of Potential siRNA Generating Region

Serine protease gene of *Acyrthosiphon pisum* (Accession number: XM_001951259.1) was downloaded from nucleotide databank of NCBI website. Qiagen siRNA design tool (Qiagen India Pvt. Ltd.) was used for identifying the region ideal for siRNA-mediated knock-down and primers were designed spanning this region using ‘Oligoperfect’ primer design tool (Invitrogen, USA). For Gateway cloning (Invitrogen, USA), attB site (25 base + 4G residues) was added at 5^/^ end of both forward and reverse primers.

### Phylogenetic Analysis of Orthologs

The orthologous sequences of serine protease from different pollinators were aligned with *MySP* sequence using Clustal W program of BioEdit software for obtaining a multiple sequence alignment file. A phylogenetic tree was constructed using the neighbor-joining method available in MEGA 4.0.2 (Mega Evolutionary Genetics Analysis) program. Bootstrap values were assessed with 1000 replicates and 64238 random seed. In addition to this, all the four sequences of Indian pollinators were aligned individually with *MySP* using bl2seq specialized BLAST available at NCBI site.

### RNA Silencing Vector and Plant Transformation

For constitution of Gateway reaction, *MySP* sequence was amplified by cDNA-PCR using a pair of gene-specific primers AttB1SP_F and AttB1SP_R, spanning +276 (GCAT) to +826 (TCGT) region on to which att sequence was added at the 5^/^-ends (Table S1). The underlined portions indicate the position of *att* sites in the primers. The purified amplicon was introduced into pDONR^TM^ 221 entry vector of Gateway system (Invitrogen, USA) by BP reaction and transformed in *E.coli* DH5α electrocompetent cells. Several recombinant clones were identified through PCR and sequenced to confirm exact insert sequence. The insert was subsequently transferred to binary vector pANDA35HK (a gift from Dr. Hiroyuki Tsuji, Nara Institute of Science and Technology, Japan) under the *CaMV35S* promoter, by LR reaction (Fig. S2A). Two copy insertion separated by a 926 bp loop was confirmed through restriction analyses (*Kpn*I-*Sac*I) and PCR using forward primer targeted to loop region of pANDA35HK vector (LOP_1F) and a reverse primer specific to *MySP* gene (SP_2R). The final RNAi binary vector was transformed in electrocompetent cells of *Agrobacterium tumeifaciens* strain GV 3101 by electroporation. Simultaneously, the *Agrobacterium* strain harboring the empty pANDA35HK vector was also prepared. Liquid broth of both the *Agrobacterium* cultures was preserved in 20% glycerol at -80°C till further use for floral dip transformation.

The floral dip transformation was carried out with the RNAi vector as well as empty vector as described in Clough and Bent [56].The transformed seeds (T_0_) of Arabidopsis were sown on MS medium in petri plates containing kanamycin [50 mg/L] for selection of transgenics. Several transgenics (SP-transgenics) were recovered with an average transformation efficiency of 5.8%. Several control plants were also generated simultaneously by transforming Arabidopsis with empty parent vector pANDA35HK without *MySP* insert. Segregation analysis of kanamycin resistance trait through successive generations of seven primary transformants (T_1_) led to the identification of T_2_ homozygous lines (SP8, SP17 and SP20) with single copy insertion. Three T_3_ plants from each of the T_2_ homozygous lines were grown to carry out subsequent molecular analyses and aphid bioassay.

### DNA, RNA and siRNA Analyses

DNA was isolated from the leaves of transgenic lines using DNAzol (Molecular Research Center, Inc., USA). PCR analyses of the genomic DNA were performed using gene-specific primers SP_1F and SP_1R. RNA was isolated using TRIzol reagent (Invitrogen, USA) and treated with DNase using TURBO DNA free (Ambion Inc., UK). For RT-PCR analyses of *MySP* and *nptII* expression in transgenics, 5 µg of total RNA was reverse transcribed using Superscript III First-Strand Synthesis System (Invitrogen, USA) and PCR amplified with ds*MySP* specific (LOP_1F, SP_2R) and *nptII* specific (Npt_1F, Npt_1R) primers respectively as described above (Table S1). In all the experiments, simultaneous amplification of tubulin as internal control ensured equal amount of cDNA in each tube. For densiometry of amplicons, integrated density values (IDV) were obtained using tools analysis application of the Alpha Innotech gel documentation system.

In transgenic lines, generation of *MySP*-specific siRNA was detected by northern blotting. Total RNA was extracted from young leaves of T_3_ plants of SP transgenics and vector transformed plants using TRIzol reagent and 50 µg total RNA was separated on 20cm long, 1.5 mm thick 15% denaturing polyacrylamide gel. The gel was run in 1X TBE buffer. The samples were mixed with equal volume of gel loading buffer II (Ambion Inc., UK) and incubated at 95°C for 5 min prior to loading onto the gel. Ultra low range RNA ladder (Fermentas Life Sciences, Canada) was also loaded onto the gel as size standard. The gels were electrophoresed at 30–45 mA constant current. The position of bromophenol blue and xylene cyanol in 15% gel corresponds to approximately 10 and 30 nt, respectively. The gel was run until the leading dye band (bromophenol blue) was near the middle of the gel. The gel was soaked for 5 min in 0.5–1 µg/ml solution of ethidium bromide in 1X TBE and washed for 2–5 min in 1X TBE. The acrylamide gel was equilibrated in 0.25X TBE buffer for 15 min and RNA was blotted to positively charged nylon membrane (Amersham Hybond ^TM^-XL, GE Healthcare Limited, USA) at 3 mA/cm^2^ and 25V for 30 minutes in 0.25X TBE using semi-dry blot apparatus (Bio-Rad Laboratories, USA). Strand-specific single-stranded RNA probe (riboprobe) was prepared by incorporation of [α-^32^P] UTP in *in vitro* transcription of PCR amplified *MySP* sequence using MAXIscript T7 kit (Ambion Inc., UK). T7 RNA polymerase promoter site was incorporated at the upstream of the reverse primer (Pro SP_R). The probe was purified to remove free nucleotides using NucAway spin columns (Ambion Inc., UK). The membrane was hybridized in ULTRAhyb-Oligo hybridization buffer (Ambion Inc., UK) overnight at 42°C with mild rotation. Following hybridization, the membrane was washed twice in 2XSSC+0.5%SDS at 42°C for 20 minutes each and exposed to X-ray film (Kodak Biomax MR Films, Sigma-Aldrich, USA) for 2 days at −80°C with an intensifying screen.

### Quantitative Real-time PCR Analyses

Real time PCR was performed to estimate siRNA-mediated down-regulation of *MySP* gene and its effect, if any, on other off-target genes in transgenic fed aphids. Aphid samples were collected from SP transgenic lines (SP8, SP17, and SP20) and vector-transformed control plants at different time points after their release. Aphid RNA isolation and cDNA preparation were carried out as described above. The cDNA was diluted 5 times to use as template in real time PCR reaction using Platinum SYBR Green qPCR SuperMix-UDG (Invitrogen, USA) on Stratagene Mx3005P (USA). For analyses of *MySP* transcript level in aphids a forward primer qSP_2F which binds 20 nt upstream of the cloned *MySP* sequence, was used along with a reverse primer qSP_2R (+63 to +86). Locations of the primers on ds*MySP* expression cassette are shown in Fig. S2A. 18S rRNA gene of *M. persicae* (Acc. No. AF487712.1) was amplified as normalizer (internal control). All reactions were run in triplicate. Relative expression levels of the genes were computed by the 2^–ΔΔCt^ method of relative quantification [57]. List of primers used for qRT-PCR of off-target genes is given in Table S1.

### Protease Assay

Synchronized nymphs of *M. persicae* (3–5 day-old) were released on control and transgenic plants and collected on 7^th^ day for protein extraction. Before release, uniformity in the developmental stage of the aphids was verified through observation under microscope and any variant was discarded. The total protein was isolated using tissue extraction reagent II (Invitrogen, USA), cleaned up and estimated as per the instructions provided with CB-X protein assay system (G-Biosciences, USA). Purified protein (10 µg) was used for protease assay using EnzChek Protease assay kit, green fluorescence (Invitrogen, USA). Fluorescence was read using Soft max Pro 5.3 software of Spectramax Gemini XS microplate reader (Molecular Devices Inc., USA). Standard curve of trypsin (Sigma-Aldrich, USA) activity was prepared independently for each replicate (*n* = 4) and the protease activity of aphid samples was expressed as equivalent to trypsin (ng/ml).

### Aphid Bioassay of Transgenic Lines

Aphid bioassay was performed on three T_3_ plants descendent from each of three independent T_2_ transgenic lines viz. SP8, SP17 and SP20. Each plant was grown in 4 inches plastic pot containing sterilized soilrite and was enclosed with a vertically placed, transparent acrylic box of dimension 6×6×18 inches so that the growth and multiplication of the released aphids could be monitored. The top of the box was covered with a glass plate. Each plant was inoculated with 10 synchronized 3–5 day-old nymphs of *M. persicae* on each of two different inflorescences and covered with a needle perforated zip lock plastic pouch. The growth, multiplication and mortality, if any, of the released aphids were monitored at regular interval and data was recorded at 8^th^ and 15^th^ day after release of aphids. The experiment was replicated for each of the three transgenic lines and vector transformed plants.

### Statistical Analysis

Data was subjected to statistical analysis by using SPSS 9.0 software. The significance of mean difference between samples at 0.05 levels was estimated by ‘Univariate analyses of Variance’ (Table S2, Table S3).

## Supporting Information

Figure S1 **Dendrogram illustrating the relatedness of predicted members of serine protease gene family in** ***A. pisum*** **with** ***MySP*** **.** Phylogenetic relationship of *MySP* and predicted members of serine protease gene family from *A. pisum* conducted using MEGA 4.0.2 is shown along with branch lengths. DNA sequences were aligned using CLUSTAL W and a tree was constructed by neighbour-joining program from a similarity matrix of pairwise comparisons. The nucleotide/ p-distance option was selected in substitution model section. Bootstrap values were assessed with 1000 replicates and are shown at the dendrogram nodes. The scale bar represents sequence divergence.
(TIF)

Figure S2 **Plant expression cassette of dsMySP and RT-PCR analysis of SP-transgenics.** (A) A diagramatic representation of the *MySP* expression cassette in RNAi vector pANDAHK::*MySP*. Different primers used for RT-PCR and qRT-PCR analyses are mapped on the loop- and *MySP-*sequence indicating their annealing positions. Names and locations of the mapped primers are indicated in the parenthesis and are as follows: 1 (LOP_1F, 582–601); 1^/^ (SP_2R, 334–353); 2^/^ (qSP_2R, 63–86); 3 (SP_1F, 172–191); 3^/^ (SP_1R, 391–410);.(B) Total RNAs (5 µg) extracted from the control and different SP transgenic lines (SP8, SP17, and SP20) were reverse transcribed, PCR amplified with either *MySP*-specific (upper panel) or *nptII*-specific (middle panel) primers and run on 2% agarose gel. Amplification of tubulin (lower panel) as internal control confirmed equal amounts of cDNA in each reaction.
(TIF)

Figure S3 **Expression analyses of off-target genes in** ***M. persicae*** **fed on SP-transgenics.** Aphids were collected from the control and SP-transgenic lines at different time points after their release for quantitative Real-time PCR analysis of the relative expression profiles of *ApSP1*, *ApSP2*, *OBP*, *CSP1* and *ACE*. 18S rRNA was used as an internal control, and values for the aphids fed on control plants were normalized to 1. Data presented are means of two independent biological replicates with three technical replicates (*n* = 6) of each ± SD. No significant difference (n.s.d) indicates that the means do not differ significantly (*P*≤0.05).
(TIF)

Table S1 **Primer sequences.**
(DOC)

Table S2 **Statistical analyses of protease assay data.**
(DOC)

Table S3 **Statistical analyses of aphid fecundity in bioassay.**
(DOC)
