# Supplementary figures and images for: Host Generated siRNAs Attenuate Expression of Serine Protease Gene in Myzus persicae
Source: PLoS One. 2012 Oct 10;7(10):e46343. doi: 10.1371/journal.pone.0046343 (PMC3468595; doi:10.1371/journal.pone.0046343)

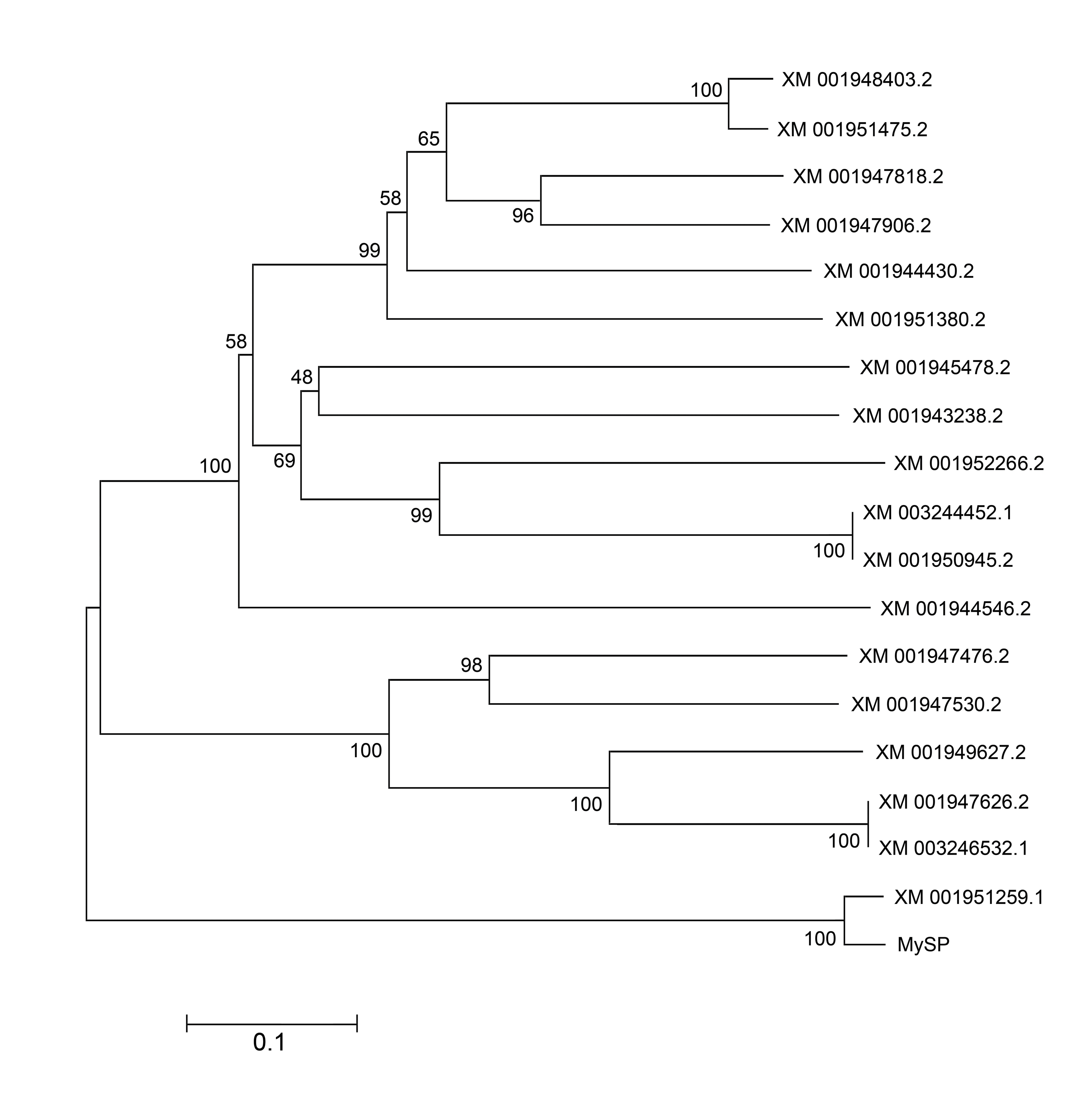

Supplement: Figure S1 — Dendrogram illustrating the relatedness of predicted members of serine protease gene family in A. pisum with MySP . Phylogenetic relationship of MySP and predicted members of serine protease gene family from A. pisum conducted using MEGA 4.0.2 is shown along with branch lengths. DNA sequences were aligned using CLUSTAL W and a tree was constructed by neighbour-joining program from a similarity matrix of pairwise comparisons. The nucleotide/ p-distance option was selected in substitution model section. Bootstrap values were assessed with 1000 replicates and are shown at the dendrogram nodes. The scale bar represents sequence divergence. (TIF) [file pone.0046343.s001.tif]

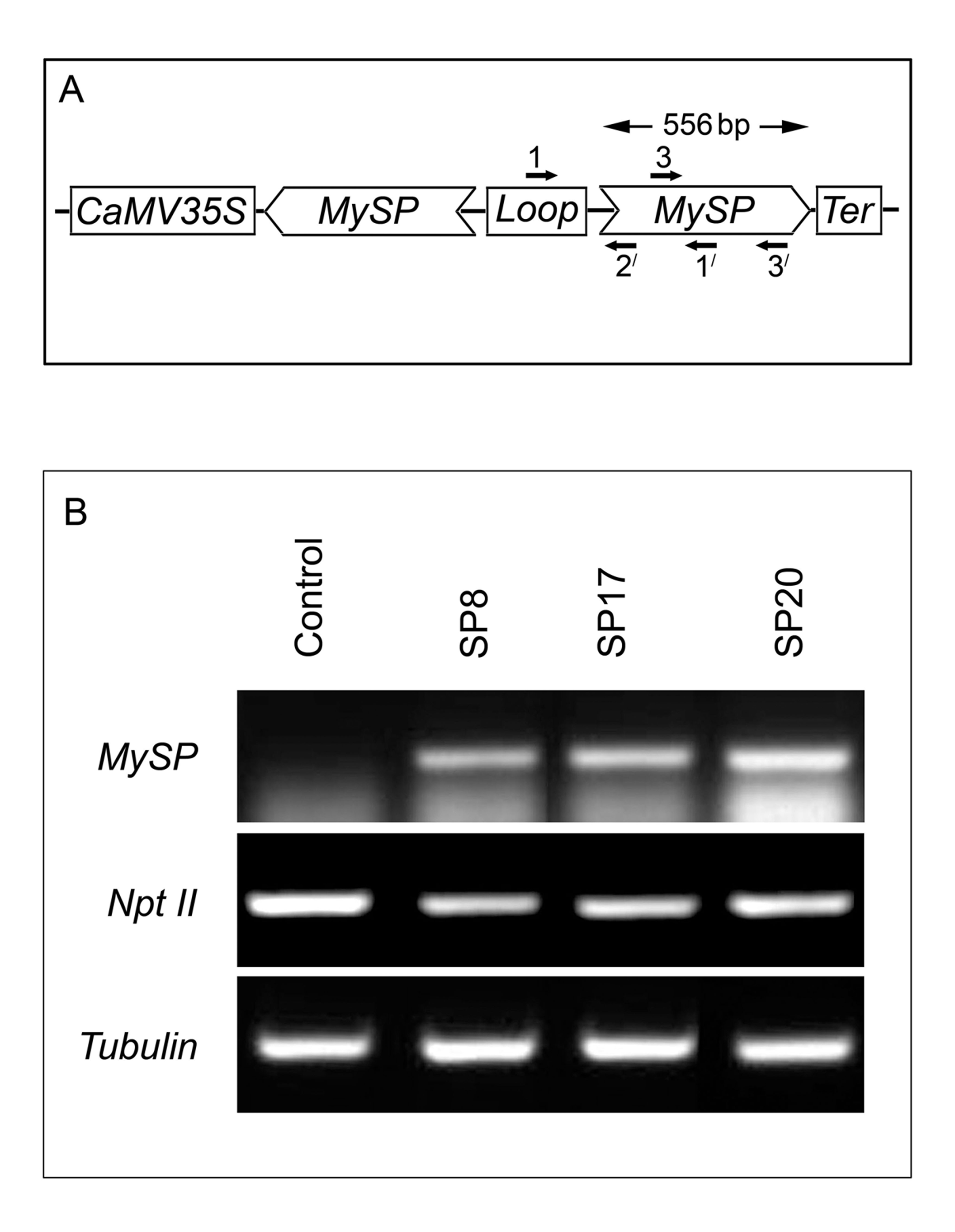

Supplement: Figure S2 — Plant expression cassette of dsMySP and RT-PCR analysis of SP-transgenics. (A) A diagramatic representation of the MySP expression cassette in RNAi vector pANDAHK::MySP. Different primers used for RT-PCR and qRT-PCR analyses are mapped on the loop- and MySP-sequence indicating their annealing positions. Names and locations of the mapped primers are indicated in the parenthesis and are as follows: 1 (LOP_1F, 582–601); 1/ (SP_2R, 334–353); 2/ (qSP_2R, 63–86); 3 (SP_1F, 172–191); 3/ (SP_1R, 391–410);.(B) Total RNAs (5 µg) extracted from the control and different SP transgenic lines (SP8, SP17, and SP20) were reverse transcribed, PCR amplified with either MySP-specific (upper panel) or nptII-specific (middle panel) primers and run on 2% agarose gel. Amplification of tubulin (lower panel) as internal control confirmed equal amounts of cDNA in each reaction. (TIF) [file pone.0046343.s002.tif]

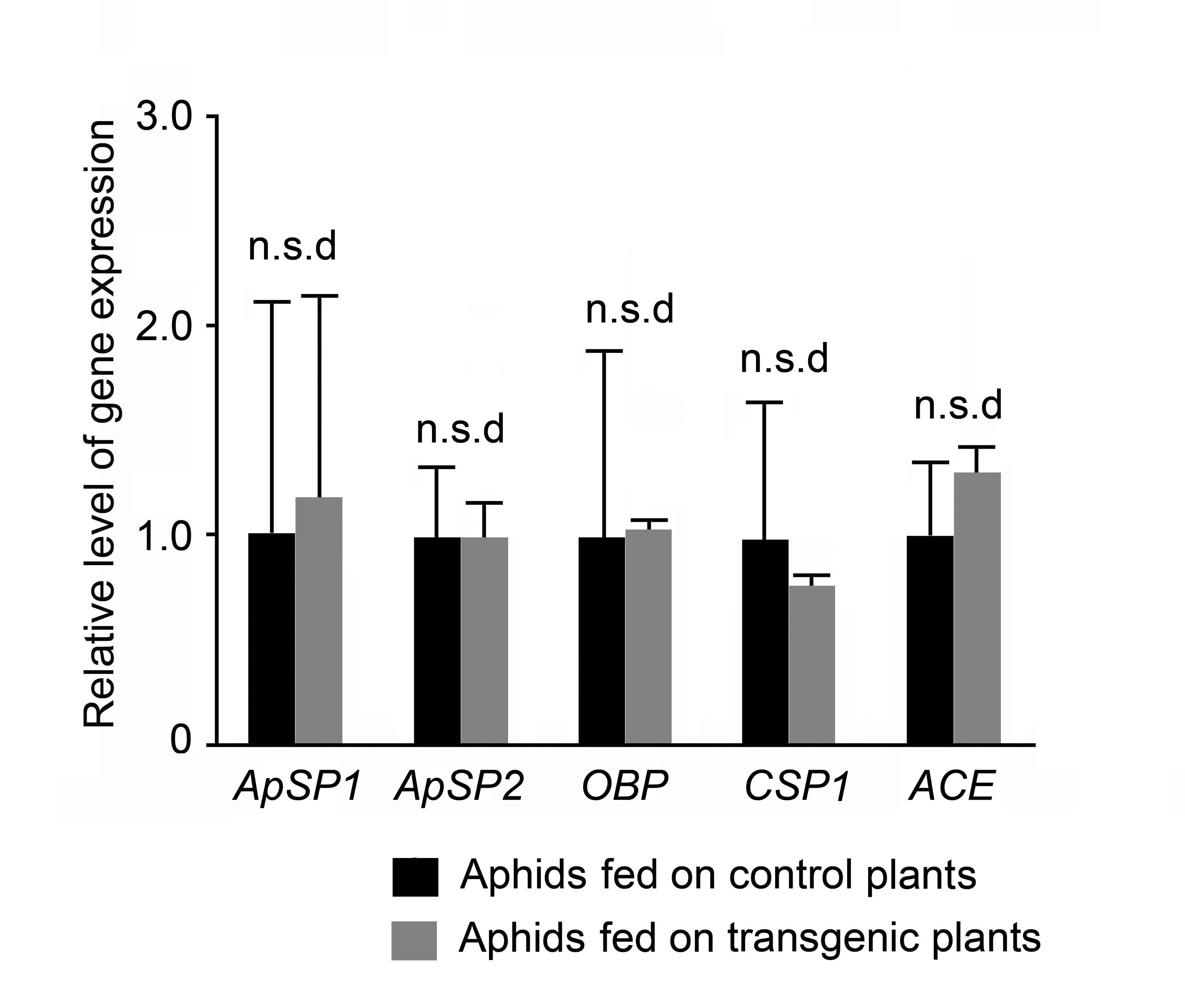

Supplement: Figure S3 — Expression analyses of off-target genes in M. persicae fed on SP-transgenics. Aphids were collected from the control and SP-transgenic lines at different time points after their release for quantitative Real-time PCR analysis of the relative expression profiles of ApSP1, ApSP2, OBP, CSP1 and ACE. 18S rRNA was used as an internal control, and values for the aphids fed on control plants were normalized to 1. Data presented are means of two independent biological replicates with three technical replicates (n = 6) of each ± SD. No significant difference (n.s.d) indicates that the means do not differ significantly (P≤0.05). (TIF) [file pone.0046343.s003.tif]
