# Supplementary material for: Host Generated siRNAs Attenuate Expression of Serine Protease Gene in Myzus persicae
Source: PLoS One. 2012 Oct 10;7(10):e46343. doi: 10.1371/journal.pone.0046343 (PMC3468595; doi:10.1371/journal.pone.0046343)
Supplement: Table S1 — Primer sequences. (DOC) [file pone.0046343.s004.doc]

**Table S1. Primer sequences.** Primers used in Gateway cloning, genomic PCR, RT-PCR and qRT-PCR experiments

| **Purpose and Primer name** | **Sequence 5/-3/** |
| --- | --- |
| **Expression analysis of *MySP***  **SP_1F**  **SP _1R**  18S rRNA_1F  18S rRNA_1R | 5/ TCCTCGGACTCCATTGTAGC 3/  5/ ACGGGCTGTGCGTAAATTAG 3/  5/atgcatgtctcagtgcaagc 3/  5/cttggatgtggtagccgttt 3/ |
| Gateway cloning of *MySP*  AttB1 SP_F  AttB2 SP_R  LOP_1F  **SP_2R** | 5/GGGGACAAGTTTGTACAAAAAAGCAGGCT- GCATTGCATTGGATATACTGATAA 3/  5/GGGGACCACTTTGTACAAGAAAGCTGGGT- ACGACGACGATGGGCCGCTG 3/  5/ TCTCTTTGATGTGCTGTGCC 3/  5/TCGTCATCGTCACACTCCAT 3/ |
| Screening of SP-transgenics  Npt_1F  Npt_1R  Tubulin_1F  Tubulin_1R | 5/ ATGTTGCTGTCTCCCAGGTC3/  5/ GCATCAGGCTCTTTCACTCC 3/  5/ CAACTCTGACCTCCGAAAGC 3/  5/ CACATTCAGCATCTGCTCGT 3/ |
| Riboprobe preparation  Pro SP_F  Pro SP_R | 5/ GCATTGCATTGGATATACTGATAAAG 3/  5/ TAATACGACTCACTATAGGGGCTGCC  GTACGACGAACT 3/ |
| Real time PCR of *MySP* RNA  qSP_2F  qSP_2R  q18S_F  q18S_R | 5/ GTCCCGAACGTCACAACA 3/  5/ AACAGGAACGAGCCCGTTGCCAAA 3/  5/ ATTCCCAGTAAGCGCGAGTCATCA 3/  5/ ACTGCGGTCGTTCAATCGGTAGTA 3/ |
| Real time PCR for off- target analyses  qApSP1_F  qApSP1_R  qApSP2_F  qApSP2_R  qACE_F  qACE_R  qOBP_F  qOBP_R  qCSP_F  qCSP_R | 5/ AGTGGAGATGATTGGAGATGCGGT 3/  5/ AGCCCAACGAGCTTTATTCCCAGA 3/  5/ AAGAGATTTGTGCTAACAGCCGCC 3/  5/ AAGCGAGCCCAACTTGCAAATCTC 3/  5/ TGTGGAACCCGAATACCAAGCTCT 3/  5/ GTATCTTGGCGTGATGCGCATTGT 3/  5/ GGCATGGAAGCGGGTTTGAAGAAA 3/  5/ ACCTCCTTCGACACAAGTAAGGCT 3/  5/ TGGTCCAGACAGCACCTGCTAAAT 3/  5/ CCAAGCGGTCGTTGTTCAGAATGT 3/ |
