# Supplementary material for: Host Generated siRNAs Attenuate Expression of Serine Protease Gene in Myzus persicae
Source: PLoS One. 2012 Oct 10;7(10):e46343. doi: 10.1371/journal.pone.0046343 (PMC3468595; doi:10.1371/journal.pone.0046343)
Supplement: Table S2 — Statistical analyses of protease assay data. (DOC) [file pone.0046343.s005.doc]

**Table S2. Statistical analyses of protease assay.**

Protease activity measured in trypsin equivalent was reduced in MySP-silenced aphids compared to the aphids fed empty vector transformed lines. Statistical analyses of trypsin-protease levels from four biological replicates were performed with Univariate ANOVA using SPSS software. The mean difference between control and transgenic lines was significant at the 0.05 level while no significant mean difference was found among the different transgenic lines.

| ***MySP*** | **Protease assay (P-value)** |
| --- | --- |
| Control – SP8 | 0.000^*^ |
| Control – SP17 | 0.000^*^ |
| Control – SP20 | 0.000^*^ |
| SP8 - SP17 | ** |
| SP17 – SP20 | ** |
| SP20 - SP8 | ** |

*. The mean difference is significant at the 0.05 level.

**. The mean difference is not significant.
