# Supplementary material for: Host Generated siRNAs Attenuate Expression of Serine Protease Gene in Myzus persicae
Source: PLoS One. 2012 Oct 10;7(10):e46343. doi: 10.1371/journal.pone.0046343 (PMC3468595; doi:10.1371/journal.pone.0046343)
Supplement: Table S3 — Statistical analyses of aphid fecundity in bioassay. (DOC) [file pone.0046343.s006.doc]

**Table S3. Statistical analyses of aphid fecundity in bioassay.**

Aphids fed on SP-transgenic *A. thaliana* plants produce significantly less progenies compared to their counterpart fed on control plants. Statistical analyses of aphid fecundity on 8^th^ and 15^th^ day after the release in six biological replicates revealed significant mean differences compared to the control-fed aphids. The mean difference between different transgenic lines was significant for 8^th^ day albeit no significant difference was found for mean values on 15^th^ day.

(Univariate ANOVA, *n=6*, P<0.05).

| ***MySP*** | **Fecundity on *A. thaliana* on 8^th^ day post-release**  **(P-value)** | **Fecundity on *A. thaliana* on 15^th^ day post-release**  **(P-value)** |
| --- | --- | --- |
| Control – SP8 | 0.000^*^ | 0.000^*^ |
| Control – SP17 | 0.000^*^ | 0.000^*^ |
| Control – SP20 | 0.000^*^ | 0.000^*^ |
| SP8 - SP17 | 0.017^*^ | ** |
| SP17 – SP20 | 0.000^*^ | ** |
| SP20 - SP8 | 0.009^*^ | ** |

*. The mean difference is significant at the 0.05 level.

**. The mean difference is not significant.
